# Ochronotic Chondropathy: A Case Report

**DOI:** 10.3390/biomedicines11102625

**Published:** 2023-09-25

**Authors:** Jake Littman, John Pietro, Jon Olansen, Chanika Phornphutkul, Roy K. Aaron

**Affiliations:** 1 Department of Orthopedic Surgery, Warren Alpert Medical School of Brown University, Providence, RI 02903, USA; 2 School of Medicine, University of Pittsburgh, Pittsburgh, PA 15261, USA; 3 Warren Alpert Medical School of Brown University, Providence, RI 02903, USA; 4 Division of Human Genetics, Department of Pediatrics, Hasbro Children’s Hospital, Warren Alpert Medical School of Brown University, Providence, RI 02903, USA

**Keywords:** ochronosis, alkaptonuria, ochronotic pigmentation, chondropathy, chondrocyte, cartilage, homogentisic acid, HGA, joint degradation

## Abstract

Endogenous ochronosis, also known as alkaptonuria, is a rare disease known for its bluish-black discoloration of the skin, sclerae, and pinnae, as well as urine that turns black upon standing. Though rarely fatal, joint degradation is a common sequela, and many patients require multiple large joint arthroplasties throughout their lifetime. Though many aspects of the pathophysiological mechanisms of the disease have been described, questions remain, such as how the initiation of ochronotic pigmentation is prompted and the specific circumstances that make some tissues more resistant to pigmentation-related damage than others. In this report, we present the case of an 83-year-old female previously diagnosed with alkaptonuria including high-quality arthroscopic images displaying the fraying of articular cartilage. We also offer a summary of the latest literature on the pathophysiological mechanisms of the disease, including cellular-level changes observed in ochronotic chondrocytes, biochemical and mechanical alterations to the cartilaginous extracellular matrix, and patterns of pigmentation and joint degradation observed in humans and mice models. With these, we present an overview of the mechanisms of ochronotic chondropathy and joint degradation as the processes are currently understood. While alkaptonuria itself is rare, it has been termed a “fundamental disease,” implying that its study and greater understanding have the potential to lead to insights in skeletal biology in general, as well as more common pathologies such as osteoarthritis and their potential treatment mechanisms.

## 1. Introduction

Ochronosis, named in reference to the Greek word “ochre” meaning “earthy” or “yellowed in appearance,” is a rare disorder that presents primarily as discolored blue-black or grey-blue pigmentation of the skin, cartilage of the ears, and sclerae [1]. Endogenous ochronosis is synonymous with alkaptonuria (AKU, OMIM 203500), which has also been called “black urine disease,” since the urine collected from those affected turns black upon standing. Clinically, in addition to the darkening of the skin, sclerae, cartilaginous structures of the ear, and urine upon exposure to air, common symptoms include shrinking intervertebral spaces, calcifications along multiple spinal regions, joint cartilage degradation, and cardiac stenosis and renal stone formation [2,3,4]. In advanced cases, the joint cartilage degradation can become so severe that complete resorption of the subchondral plate and the total destruction of the joint can be observed [5]. Skin and cartilaginous structure discoloration can often present later in life, while arthralgia and back pain often present earlier with varying severity [1]. Though rarely fatal, AKU is a progressive lifelong condition that affects patients’ quality of life, often causing severe joint pain and necessitating multiple large joint arthroplasties.

AKU is believed to be caused by a rare autosomal recessive genetic variant of the homogentisate dioxygenase gene, *HGD*, which is located on chromosome 3q21–23 [6]. The biochemical diagnosis of AKU is made through the detection of homogentisic acid (HGA) in the urine, and the molecular diagnosis is based upon the identification of pathogenic variants of *HGD*, the gene responsible for producing the enzyme homogentisate 1,2-dioxygenase (HGD) [7]. Variants found to be implicated in the disease include frameshift, missense, nonsense, and splicing [8]. It was the first human disorder shown to follow Mendelian inheritance [9]. Current literature suggests that the inactivation of the *HGD* gene arrests the production of HGD, which is a key component of phenylalanine and tyrosine catabolism. Without HGD, these amino acids cannot be fully degraded, resulting in a buildup of excess HGA, an intermediate of their catabolic pathway [10]. Current understanding suggests that HGA oxidizes to benzoquinone acetic acid before irreversibly decaying into ochronotic pigment which can be found bound to collagen, compromising its structural integrity and giving it a bluish tint. As discussed later in this review, it is currently unknown whether HGA itself, its oxidized intermediate benzoquinone acetic acid, or ochronotic pigment initially binds to collagen fibrils [11]. Evidence suggests that these compounds play individualized roles dependent upon tissue and bodily location, providing some explanation for the diverse multi-system involvement ochronosis displays [1,12].

There are two classifications of ochronosis, endogenous and exogenous, with differing conditions that precipitate them. Both present in similar ways, and both are thought to be derived from a buildup of HGA. However, exogenous ochronosis is often less severe in presentation and only results in cartilage discoloration. The key difference between the two diseases involves how the buildup of HGA is triggered; in exogenous ochronosis, the buildup of acid is acquired via the inhibition of HGD from an outside influence rather than a genetic mutation, often a side effect of drugs like acne medications such as minocycline, or potentially hydroquinone which has utilizations in both medications and cosmetics [13,14,15]. 

AKU, or endogenous ochronosis, is rare, with global prevalence estimated to lie somewhere between 1/100,000 to 1/250,000 and the prevalence in the United States estimated to be 1 per 1,000,000 [16,17]. Like many rare diseases, however, its prevalence can sharply rise in specific populations. In particular, South Africa, the Dominican Republic, and Slovakia have all been shown to harbor populations at greater risk [18]. Exogenous ochronosis is markedly more common than endogenous, especially in areas where phenol-containing prescriptions are routine. For instance, many antimalarials are comprised of such compounds, resulting in an uptick in exogenous ochronosis prevalence where malaria is endemic [13,18].

Investigations into the pathogenic mechanisms of AKU have opened the door to a greater understanding of much more common diseases like osteoarthritis. AKU has been deemed a “fundamental disease,” described as “rare genetic disorders that are gateways to understanding common conditions and human physiology” [19]. Shared aspects of ochronosis and osteoarthritis include articular cartilage degeneration, osteophyte formation, synovial inflammation, thickening of the subchondral bone, dysregulation of cellular signaling pathways including Hedgehog signaling, cell death of chondrocytes via chondroptosis, and the appearance of high-density mineralized protrusions [20,21]. Furthermore, recent investigations applying nuclear magnetic resonance (NMR) spectroscopy to the examination of AKU cartilage and osteoarthritic cartilage found similar intrastrand disruption of the collagen triple helix in both [22]. A greater understanding of how ochronosis leads to chondropathy and cartilage damage can lead to a better understanding of a variety of disease pathologies and potential treatments, both for patients suffering from ochronosis, as well as those suffering from much more common conditions like osteoarthritis.

## 2. Case Report

**Figure 1:**
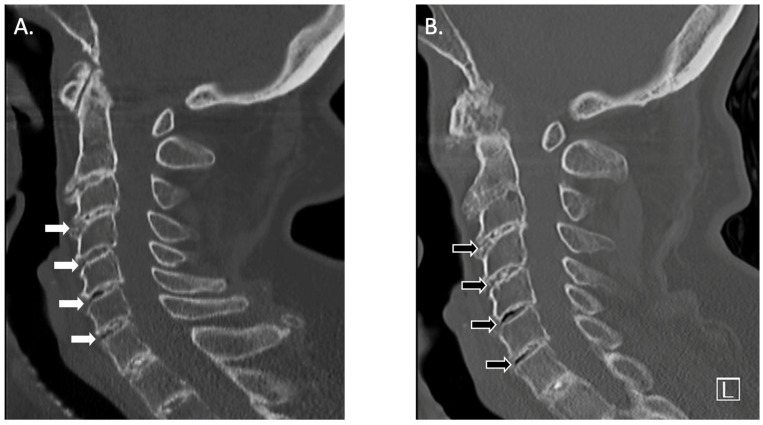
(**A**) Sagittal computed tomogram of the cervical spine of the patient taken 6 years ago when she was 77 years old. Destruction of intervertebral disc cartilage is apparent (white arrows). (**B**) Sagittal computed tomogram of the cervical spine of the same patient five years later, when she was 82 years old, showing progressive disc degeneration (black arrows) resulting from advancing disc cartilage destruction and reactive bone formation over the 5-year period. Vacuum phenomena are also apparent in the inferior two disc spaces.

**Figure 2:**
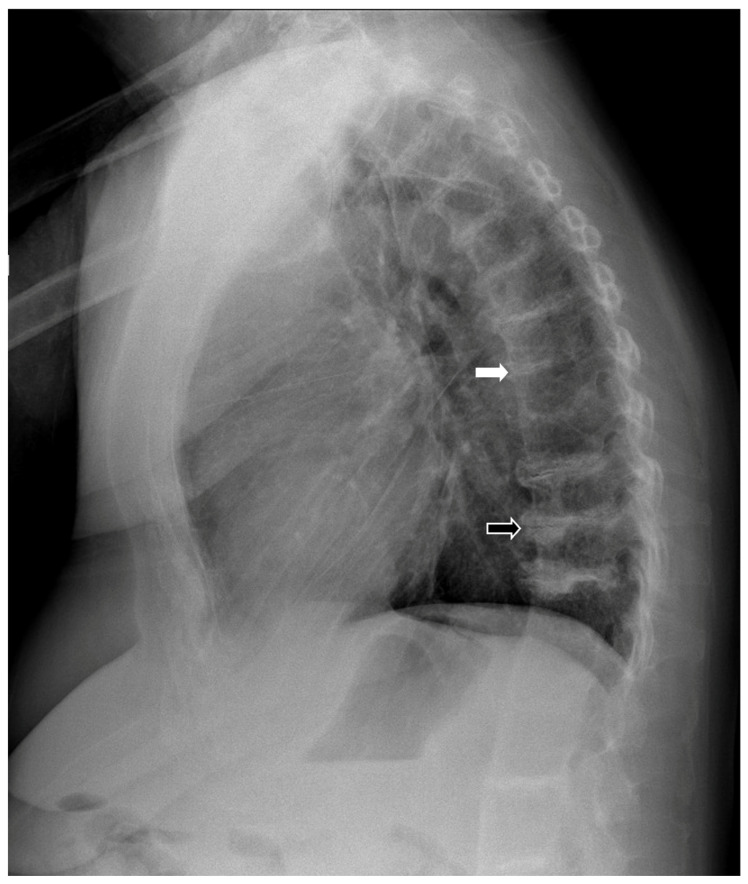
Lateral chest X-ray of the patient taken 2 years ago when she was 81 years old. The spine exhibits degeneration of the intervertebral disc cartilage with end-plate sclerosis (white arrow) and osteophytes typical of lumbar spondylosis (black arrow). The intervertebral bodies may be osteoporotic.

**Figure 3:**
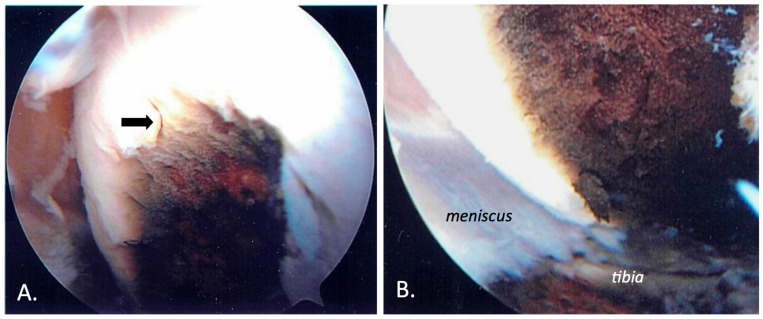
Arthroscopic images obtained during the patient’s arthroscopy 23 years earlier, when she was 60 years old, revealing ochronosis of the medial femoral condyle with brownish-black discoloration. (**A**) Damage to the superficial articular surface can be seen (arrow). (**B**) Destruction of the deeper layers of articular cartilage is evident resulting in an insufficient bearing surface.

**Figure 4:**
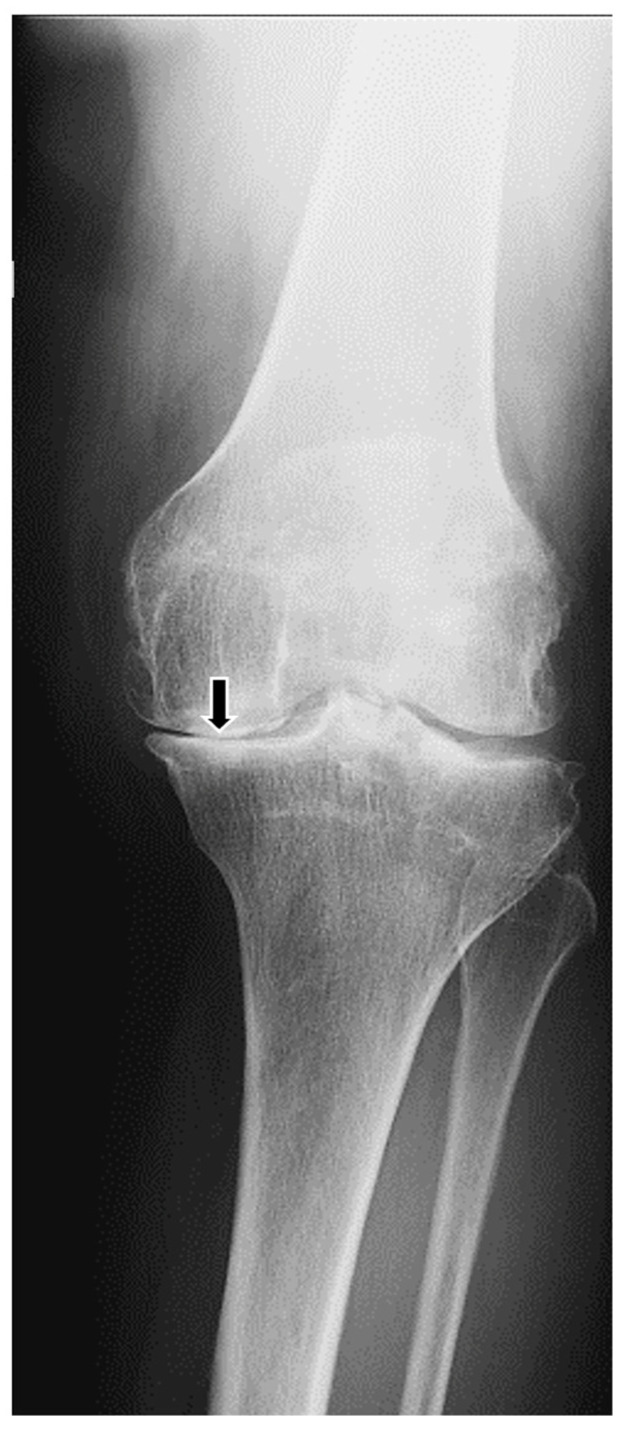
Anteroposterior radiograph of the patient’s knee taken 14 years ago when she was 69 years old. The medial tibiofemoral compartment articular cartilage has been destroyed (black arrow) and reactive bone and osteophytes have formed. Varus knee angulation has resulted. The radiographic appearance is typical of osteoarthritis which can result from a variety of chondral insults including genetic, inflammatory, septic, and mechanical conditions.

We report the case of an 83-year-old female presenting with a multi-decade history of ochronosis. On the initial orthopedic evaluation, she complained of back and joint pains. A history of dark urine upon standing since childhood and progressive discoloration of her sclerae and pinnae were noted, consistent with her prior clinical diagnosis of AKU. She denied taking medications known to cause cartilage discoloration. Initial physical examination and radiographic imaging were consistent with early multifocal osteoarthritis and extensive degenerative disc disease. She was previously followed with a multi-year history of progressive spine, hip, and knee pain. CT scans of her spine 5 years apart show progressive loss of intervertebral disc cartilage (Figure 1). Subsequent X-ray imaging demonstrated progressive degenerative spondylosis (Figure 2). After being diagnosed, she was treated for about 20 years with conservative joint-preserving measures, including oral non-steroidal anti-inflammatory drugs (NSAIDs), intra-articular corticosteroids, and physical therapy. Eventually, at the age of 60, she underwent knee arthroscopy, at which time findings included brown/black discoloration of articular cartilage, erosion of superficial zones of articular cartilage, and fraying and destruction of deeper cartilage layers (Figure 3). Although the pathology was revealed, the procedure did not result in substantial pain relief because of an incompetent cartilage-bearing surface. Eventually, her right knee pain became too severe to be managed non-operatively. X-rays showed generalized chondrolysis and loss of the medial tibiofemoral joint space, as well as reactive subchondral bone and osteophytes characteristic of osteoarthritis (Figure 4). At the age of 68, she underwent a successful right total knee replacement, followed in succession 2 years later by a right hip replacement and 3 years after that by a left knee replacement, all due to loss of articular cartilage, secondary osteoarthritis, and unremitting and unmanageable joint pain. She has experienced chronic back pain and kyphosis; however, the pain is well-controlled with NSAIDs and she continues to live independently.

## 3. Pathophysiology

As discussed throughout this paper, much work has been conducted to understand the pathophysiology of ochronosis. However, pertinent questions still remain, including those related to the molecular mechanisms of the disease and what influences its onset and progression [23]. Mouse models have been useful in studying the pathophysiologic mechanisms of the disease, but factors including the relatively short lifespan of mice when compared to humans and differences in joint loading and cellular turnover rates limit the correlations from these studies to human disease processes [24].

In humans, ochronotic pigment tends to appear initially in areas of high mechanical loading such as the weight-bearing joints [5,19,23]. Ochronotic pigment has been shown to cause micro- and macro-level changes, ranging from biochemical and biomechanical disturbances in individual chondrocytes to grossly observed pigmentation and degradation of ochronotic cartilage. In one study, it was discovered that the activity of Tergazyme, a detergent with protease enzyme that “effectively digests noncalcified ECM”, was unable to decompose ochronotic cartilage [5]. This finding, along with the fact that acidification of urine turned black due to AKU does not restore its original color, provides evidence that the deposit of ochronotic pigmentation is potentially irreversible [11].

We structure our review of the current literature on ochronotic chondropathy as follows. First, biochemical and biomechanical disturbances which can be observed in individual chondrocytes are discussed. Second, we examine the literature surrounding how the extracellular matrix (ECM) of cartilage is affected, including alterations in ECM composition and how ochronotic pigmentation accumulates on collagen. Lastly, we discuss reports examining the larger-scale patterns of ochronotic pigmentation observed in mouse models of AKU and those extracted from human patients during joint replacement surgery. Combining the findings from each of these sections, we are presented with a comprehensive overview of the latest literature on how ochronosis damages joints, from the level of individual chondrocytes to grossly observable changes in cartilage ultimately leading to degradation and necessitating surgical intervention. 

### 3.1. Cellular-Level Changes in Chondrocytes

Chondrocytes, as the only cells found in cartilage [25], play a crucial role in the pathogenesis of ochronosis. One report showed that in vitro, an HGA-treated medium without cells took three weeks to darken to the same degree that HGA-treated medium with cells achieved in three days [26], suggesting that cells play a crucial role in accelerating the process of pigmentation deposition. A growing body of research has shed light on a variety of biochemical and biomechanical changes at the level of individual chondrocytes in the setting of HGA. In 2021, Galderisi et al. published a study in which they used a model of AKU chondrocytes to examine the effects of HGA on the chondrocyte cytoskeleton and found distinct alterations in the concentration and organization of cytoskeletal proteins when compared to control chondrocytes [27]. More specifically, they found reduced concentrations of actin, vimentin, and tubulin with associated microstructural disorganization of all three in chondrocytes exposed to HGA compared to controls [27].

In 2016, Gambassi et al. reported that chondrocytes treated with HGA had shorter primary cilia and dysregulated Hedgehog signaling when compared to control chondrocytes [20]. Hedgehog signaling plays a role in the regulation of chondrocyte growth and differentiation, and increased Hedgehog signaling has been implicated in osteoarthritis [28]. Primary cilia have previously been described as “a master regulator of cell signaling” and have been shown to be involved in inflammatory signaling in chondrocytes [29]. Recent work has suggested that in the setting of inflammation, the intracellular metabolic processes of chondrocytes can be altered, leading to the expression of ECM-degrading enzymes in a process known as metabolic reprogramming [30]. Interestingly, Gambassi et al. reported that when HGA-treated chondrocytes were introduced to antagonists of Smoothened, a receptor-like protein found on primary cilia, cilia length was restored to normal levels, thus outlining a potential treatment mechanism for AKU in need of further investigation [20].

Another recent report showed that human chondrocytes exposed to chronic treatment of HGA experienced decreased levels of autophagic processes and the accumulation of ochronotic pigmentation, leading to the proposition that the former is responsible for the latter [31]. Chondroptosis, a distinct form of cell death of chondrocytes that has been implicated in the settings of osteoarthritis, trauma, and hyperthermia [32,33,34], was also observed in that experiment, as was mitochondrial damage [31]. Chondroptosis and classical apoptosis share a variety of similarities and differences, but one major distinction between them is the reliance on autophagocytosis in chondroptosis, rather than heterophagocytosis by phagocytes as seen in classical apoptosis [32,33]. This process of autophagocytosis provides a potential explanation for the empty lacunae sometimes observed in AKU cartilage [35].

Taken together, the current literature on ochronotic chondrocytes suggests that key pathophysiological features may include (1) cytoskeletal disorganization; (2) dysregulation of cell signaling processes (including Hedgehog signaling); (3) shortened cilia length; (4) decreased autophagic processes facilitating the accumulation of intracellular pigmentation and (5) increased levels of cell death via chondroptosis.

### 3.2. Alterations to the Extracellular Matrix

**Figure 5:**
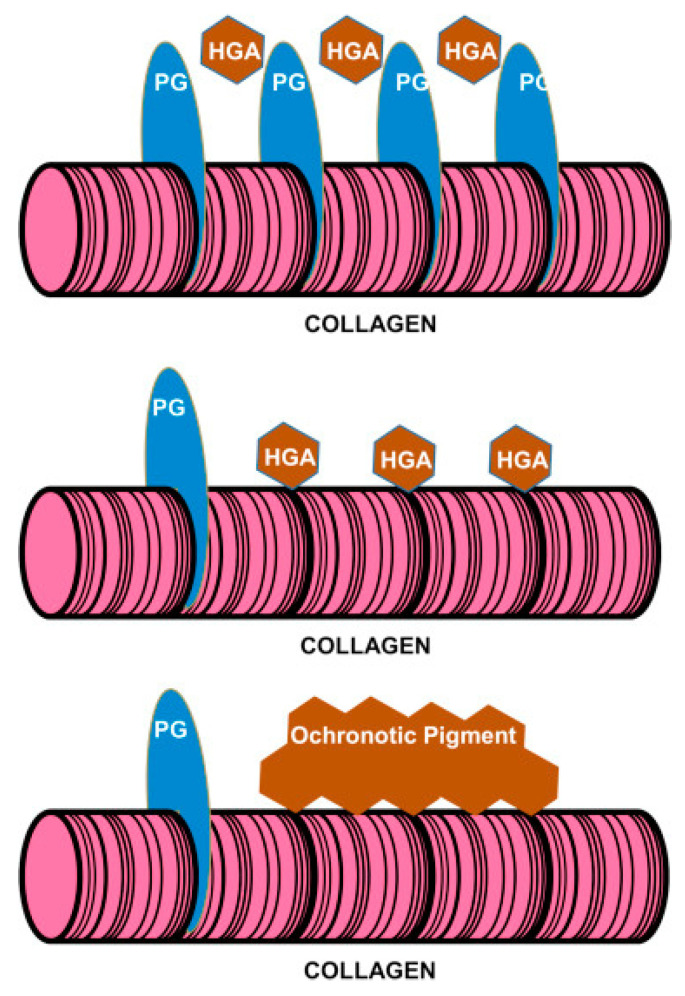
Diagrammatic representation of the exposed collagen hypothesis as described by Gallagher et al. The top panel shows collagen in its native state, surrounded by a protective layer of proteoglycans (PGs) disallowing the binding of HGA. The middle panel shows the periodic binding of HGA after protective PGs have been lost from collagen due to mechanical loading, aging, degeneration, or some other insult. The bottom panel shows the deposition of ochronotic pigment onto the exposed collagen, making it stiffer and leading to a downward spiral of further pigmentation and damage. Note that while the middle panel displays HGA itself binding the collagen, it is not currently known whether it is HGA, its oxidized intermediate benzoquinone acetic acid, or ochronotic pigment that first binds to collagen. Adapted with permission from Ref. [19]. 2016, *Seminars in Cell & Developmental Biology*.

While the mechanisms through which excess circulating HGA leads to the onset and progression of ochronosis are still being elucidated [23], there is a growing body of prior work offering insights into these questions. In 2010, an ultrastructural examination of ochronotic tissue revealed two distinct forms of extracellular pigmentation; a periodicity of minute ochronotic granules was seen on some collagen fibers, and larger, crystal-like pigmentation structures were observed entirely encasing other fibers [36]. From these observations, it was proposed that the former structure may precipitate the latter, i.e., the minute periodic granules may act as nucleation points for the further polymerization of ochronotic pigment into larger crystal-like structures covering collagen [36]. This work was one of the foundations for what Gallagher et al. described as the “exposed collagen hypothesis” which includes the following principles: (1) “there are specific sites on collagen where HGA can bind but which are protected in native collagen in undamaged extracellular matrix”; (2) “following structural and compositional changes, including loss of PGs, the potential binding sites become exposed allowing HGA to bind”; (3) “binding of HGA-derived pigment to the collagen fibres makes them stiffer and susceptible to more mechanical damage”; (4) “this leads to further ultrastructural changes in collagen, increased exposure of binding sites to HGA and a downward spiral of pigmentation” (Figure 5) [19]. While these descriptions refer to the binding of HGA and HGA-derived pigment to the collagen fibrils, it is important to note that it is not currently known whether it is HGA itself, its oxidized intermediate, benzoquinone acetic acid, or ochronotic pigment that initially binds to collagen fibrils [11]. Furthermore, though the pigmentation that is characteristic of ochronosis is often described as a “polymer”, the notion that these structures are produced by the association of multiple identical monomers (i.e., by homopolymerization) has been contested, and it has been noted that nonpolymeric structures can also produce dark pigmentation similar to ochronosis [37].

As discussed earlier, Galderisi et al. found differences in the concentrations and organization of actin, vimentin, and tubulin in HGA-exposed chondrocytes compared to controls [27]. These findings led to the proposition that these changes play a role in the impaired synthesis and excretion of ECM components observed in ochronosis [27]. Other literature has supported this hypothesis; a study examining cartilage matrix components from 0.6% of the total global population of AKU patients reported a lower turnover state, accelerated aging, greater total extractable protein, and lower levels of extractable glycosaminoglycans in AKU cartilage compared to samples from osteoarthritic and healthy (non-arthritic) patients [38]. Another study examining human ochronotic articular cartilage found increased porosity, decreased water content, and decreased heat capacity when compared to healthy cartilage, as well as associated alterations in rheologic capacity as measured by strain sweep test, oscillatory sheer stress analysis, and stress-relaxation test [39]. From these studies, it is clear that (1) ochronotic pigment deposition is involved in the disturbance of ECM homeostasis, and (2) these disturbances result in alterations to the rheologic capacity of joints, potentially leading to a downward spiral further inducing mechanical degradation and leading to the destruction of the joint.

### 3.3. Patterns of Structural Damage Observed in Cartilage 

**Figure 6:**
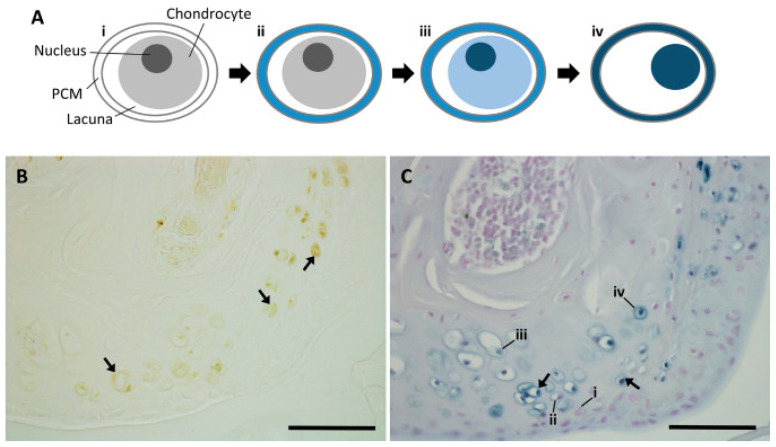
From Hughes et al.: Progression of ochronotic pigment in articular cartilage in a 66-week-old BALB/c *Hgd^−/−^* mouse. (**A**) A diagram displaying the progression of ochronotic pigmentation as observed in chondrocytes in the region of articular calcified cartilage. (**i**) A healthy, unpigmented chondrocyte; (**ii**) chondrocyte displaying pericellular pigmentation, the initial pigmentation to be observed; (**iii**) chondrocyte displaying progression to intracellular pigmentation as is typically observed after pericellular pigmentation; (**iv**) chondrocyte displaying more dramatic intracellular pigmentation and associated pyknosis. (**B**) Chondrocytes of the medial femoral condyle of the knee displaying ochronotic pigmentation (arrows) as observed without staining. (**C**) Chondrocytes in the articular calcified cartilage displaying the four steps of pigmentation (**i**–**iv**) as described in the diagram in (**A**), observed with Schmorl’s staining. Scale bar in (**B**,**C**) = 50 µm. Adapted with permission from Ref. [40]. 2021, *Calcified Tissue International*.

Hughes et al. used AKU mouse models to study the anatomical distribution of ochronotic pigmentation and determine which tissues are the primary sites to become susceptible to pigmentation [40]. Amongst their findings, they observed that (1) the site of initial pigment deposition was associated with individual chondrocytes in the calcified cartilage (Figure 6), consistent with other prior research examining both mouse models of AKU and human AKU patients [5,24], and (2) areas under greater physiological load, such as in the lumbar vertebral endplates compared to those at the base of the tail, displayed greater numbers of pigmented chondrocytes, also consistent with previous research [5,40].

Vigorita et al. collected and stained samples of intact articular cartilage from a 73-year-old woman who underwent total knee replacement secondary to advanced ochronosis, leading to some of the most striking images of advanced ochronotic pigmentation taken from a human being collected to date [41]. In their report, blanket pigmentation was observed in both the radial and transitional zones, though it was most prominent in the radial zone where it appeared both intracellularly in chondrocytes and within the ECM, while pigmentation was absent in the superficial zone and calcified zone except for some relatively minor pericellular pigmentation in the calcified zone [41]. Based on these results, they hypothesized that (1) the avascularity of cartilage generally disallows the clearing of HGA prior to polymerization and therefore facilitates the deposition of the ochronotic pigment; (2) the movement of water between the superficial zone and the synovial fluid clears HGA, accounting for the relative lack of pigmentation in the superficial zone; and (3) the relatively low metabolic activity and turnover rate of chondrocytes in the calcified zone lead to a low production rate of HGA, thus accounting for the relative lack of pigmentation compared to the radial and transitional zones in advanced ochronotic cartilage [41].

## 4. Conclusions

Much prior work has been conducted examining the processes leading to, and the effects of, ochronotic pigmentation. Breakthroughs from these efforts have elucidated ochronitc pigmentation’s effects on individual chondrocytes, the ECM of cartilage, and patterns of damage observed throughout affected joints. These insights include the propositions that (1) individual chondrocytes affected by pigmentation undergo dysregulations in cytoskeletal concentration and organization, cell signaling, organelle functioning including mitochondrial and cilia functioning, and cell death via chondroptosis; (2) ECM is initially resistant to pigmentation but undergoes a positive-feedback-like process through which collagen damage leads to initial periodicities of ochronotic pigmentation that act as nucleation points for further pigment deposition; and (3) ochronotic pigmentation is primarily seen in the pericellular area of chondrocytes in articular calcified cartilage before moving intracellularly and eventually spreading to the radial and transitional zones, leading to the potential resorption of the subchondral bone plate and total destruction of the joint. While AKU is rare, it is a debilitating disease that often leads to multiple large joint arthroplasties as demonstrated by the case reported here. Its proposed status as a “fundamental disease” dictates that insights into its pathophysiological processes have the possibility of shedding light on other more common diseases like osteoarthritis. This notion has proven fruitful, as illustrated through the discovery of mechanisms related to both diseases such as chondroptosis and the formation of high-density mineralized protrusions. Further study may lead to more insights, paving the way to potential treatment mechanisms for both diseases and a greater understanding of skeletal biological processes.

## Data Availability

No new data were created or analyzed in this study. Data sharing is not applicable to this article.

## References

[1] Phornphutkul C., Introne W.J., Perry M.B., Bernardini I., Murphey M.D., Fitzpatrick D.L., Anderson P.D., Huizing M., Anikster Y., Gerber L.H. (2002). Natural history of alkaptonuria. N. Engl. J. Med..

[2] Selvi E., Manganelli S., Mannoni A., Benucci M., Minacci C., Marcolongo R. (2000). Chronic ochronotic arthritis: Clinical, arthroscopic, and pathologic findings. J. Rheumatol..

[3] Hamdi N., Cooke T.D.V., Hassan B. (1999). Ochronotic arthropathy: Case report and review of the literature. Int. Orthop..

[4] Laskar F.H., Sargison K.D. (1970). Ochronotic arthropathy: A review with four case reports. J. Bone Jt. Surg. Br..

[5] Taylor A.M., Boyde A., Wilson P.J.M., Jarvis J.C., Davidson J.S., Hunt J.A., Ranganath L.R., Gallagher J.A. (2011). The role of calcified cartilage and subchondral bone in the initiation and progression of ochronotic arthropathy in alkaptonuria. Arthritis Rheum..

[6] Vilboux T., Kayser M., Introne W., Suwannarat P., Bernardini I., Fischer R., O’Brien K., Kleta R., Huizing M., Gahl W.A. (2009). Mutation spectrum of homogentisic acid oxidase (*HGD*) in alkaptonuria. Hum. Mutat..

[7] Introne W.J., Perry M., Chen M., Adam M.P., Mirzaa G.M., Pagon R.A., Wallace S.E., Bean L.J.H., Gripp K.W., Amemiya A. (1993). Alkaptonuria. GeneReviews^®^.

[8] Zatkova A. (2011). An update on molecular genetics of Alkaptonuria (AKU). J. Inherit. Metab. Dis..

[9] Garrod A.E. (1996). The incidence of alkaptonuria: A study in chemical individuality. Mol. Med..

[10] Zatkova A., Sedlackova T., Radvansky J., Polakova H., Nemethova M., Aquaron R., Dursun I., Usher J.L., Kadasi L. (2011). Identification of 11 Novel Homogentisate 1,2 Dioxygenase Variants in Alkaptonuria Patients and Establishment of a Novel LOVD-Based HGD Mutation Database. JIMD Reports-Case and Research Reports, 2012/1.

[11] Ranganath L.R., Norman B.P., Gallagher J.A. (2019). Ochronotic pigmentation is caused by homogentisic acid and is the key event in alkaptonuria leading to the destructive consequences of the disease—A review. J. Inherit. Metab. Dis..

[12] Tharini G., Ravindran V., Hema N., Prabhavathy D., Parveen B. (2011). Alkaptonuria. Indian J. Dermatol..

[13] Bruce S., Tschen J.A., Chow D. (1986). Exogenous ochronosis resulting from quinine injections. J. Am. Acad. Dermatol..

[14] Levin C.Y., Maibach H. (2001). Exogenous ochronosis. An update on clinical features, causative agents and treatment options. Am. J. Clin. Dermatol..

[15] Findlay G.H., Morrison J.G., Simson I.W. (1975). Exogenous ochronosis and pigmented colloid milium from hydroquinone bleaching creams. Br. J. Dermatol..

[16] Sharabi A.F., Goudar R.B. (2023). Alkaptonuria. StatPearls.

[17] Mistry J.B., Bukhari M., Taylor A.M. (2013). Alkaptonuria. Rare Dis..

[18] Bhattar P.A., Zawar V.P., Godse K.V., Patil S.P., Nadkarni N.J., Gautam M.M. (2015). Exogenous Ochronosis. Indian J. Dermatol..

[19] Gallagher J.A., Dillon J.P., Sireau N., Timmis O., Ranganath L.R. (2016). Alkaptonuria: An example of a “fundamental disease”—A rare disease with important lessons for more common disorders. Semin. Cell Dev. Biol..

[20] Gambassi S., Geminiani M., Thorpe S.D., Bernardini G., Millucci L., Braconi D., Orlandini M., Thompson C.L., Petricci E., Manetti F. (2017). Smoothened-antagonists reverse homogentisic acid-induced alterations of Hedgehog signaling and primary cilium length in alkaptonuria. J. Cell. Physiol..

[21] Gallagher J.A., Dillon J.P., Ranganath L.R. (2021). Development of an Effective Therapy for Alkaptonuria—Lessons for Osteoarthritis. Rheumatol. Immunol. Res..

[22] Chow W.Y., Norman B.P., Roberts N.B., Ranganath L.R., Teutloff C., Bittl R., Duer M.J., Gallagher J.A., Oschkinat H. (2020). Pigmentation Chemistry and Radical-Based Collagen Degradation in Alkaptonuria and Osteoarthritic Cartilage. Angew. Chem. Int. Ed..

[23] Zatkova A., Ranganath L., Kadasi L. (2020). Alkaptonuria: Current Perspectives. Appl. Clin. Genet..

[24] Preston A.J., Keenan C.M., Sutherland H., Wilson P.J., Wlodarski B., Taylor A.M., Williams D.P., Ranganath L.R., A Gallagher J., Jarvis J.C. (2014). Ochronotic osteoarthropathy in a mouse model of alkaptonuria, and its inhibition by nitisinone. Ann. Rheum. Dis..

[25] Liu Y., Zhang Z., Li T., Xu H., Zhang H. (2022). Senescence in osteoarthritis: From mechanism to potential treatment. Arthritis Res. Ther..

[26] Tinti L., Taylor A.M., Santucci A., Wlodarski B., Wilson P.J., Jarvis J.C., Fraser W.D., Davidson J.S., Ranganath L.R., Gallagher J.A. (2011). Development of an in vitro model to investigate joint ochronosis in alkaptonuria. Rheumatology.

[27] Galderisi S., Cicaloni V., Milella M.S., Millucci L., Geminiani M., Salvini L., Tinti L., Tinti C., Vieira O.V., Alves L.S. (2021). Homogentisic acid induces cytoskeleton and extracellular matrix alteration in alkaptonuric cartilage. J. Cell. Physiol..

[28] Lin A.C., Seeto B.L., Bartoszko J.M., Khoury M.A., Whetstone H., Ho L., Hsu C., Ali S.A., Alman B.A. (2009). Modulating hedgehog signaling can attenuate the severity of osteoarthritis. Nat. Med..

[29] Wann A.K., Chapple J.P., Knight M.M. (2014). The primary cilium influences interleukin-1β-induced NFκB signalling by regulating IKK activity. Cell. Signal..

[30] Arra M., Abu-Amer Y. (2023). Cross-talk of inflammation and chondrocyte intracellular metabolism in osteoarthritis. Osteoarthr. Cartil..

[31] Galderisi S., Milella M.S., Rossi M., Cicaloni V., Rossi R., Giustarini D., Spiga O., Tinti L., Salvini L., Tinti C. (2022). Homogentisic acid induces autophagy alterations leading to chondroptosis in human chondrocytes: Implications in Alkaptonuria. Arch. Biochem. Biophys..

[32] Roach H.I., Aigner T., Kouri J.B. (2004). Chondroptosis: A variant of apoptotic cell death in chondrocytes?. Apoptosis.

[33] Salucci S., Falcieri E., Battistelli M. (2022). Chondrocyte death involvement in osteoarthritis. Cell Tissue Res..

[34] Battistelli M., Salucci S., Olivotto E., Facchini A., Minguzzi M., Guidotti S., Pagani S., Flamigni F., Borzì R.M., Falcieri E. (2014). Cell death in human articular chondrocyte: A morpho-functional study in micromass model. Apoptosis.

[35] Millucci L., Giorgetti G., Viti C., Ghezzi L., Gambassi S., Braconi D., Marzocchi B., Paffetti A., Lupetti P., Bernardini G. (2015). Chondroptosis in Alkaptonuric Cartilage. J. Cell. Physiol..

[36] Taylor A.M., Wlodarski B., Prior I.A., Wilson P.J.M., Jarvis J.C., Ranganath L.R., Gallagher J.A. (2010). Ultrastructural examination of tissue in a patient with alkaptonuric arthropathy reveals a distinct pattern of binding of ochronotic pigment. Rheumatology.

[37] Roberts N.B., Curtis S.A., Milan A.M., Ranganath L.R. (2015). The Pigment in Alkaptonuria Relationship to Melanin and Other Coloured Substances: A Review of Metabolism, Composition and Chemical Analysis. JIMD Rep..

[38] Taylor A.M., Hsueh M.-F., Ranganath L.R., Gallagher J.A., Dillon J.P., Huebner J.L., Catterall J.B., Kraus V.B. (2017). Cartilage biomarkers in the osteoarthropathy of alkaptonuria reveal low turnover and accelerated ageing. Rheumatology.

[39] Bernardini G., Leone G., Millucci L., Consumi M., Braconi D., Spiga O., Galderisi S., Marzocchi B., Viti C., Giorgetti G. (2019). Homogentisic acid induces morphological and mechanical aberration of ochronotic cartilage in alkaptonuria. J. Cell. Physiol..

[40] Hughes J.H., Keenan C.M., Sutherland H., Edwards H.R., Wilson P.J.M., Ranganath L.R., Jarvis J.C., Bou-Gharios G., Gallagher J.A. (2021). Anatomical Distribution of Ochronotic Pigment in Alkaptonuric Mice is Associated with Calcified Cartilage Chondrocytes at Osteochondral Interfaces. Calcif. Tissue Int..

[41] Vigorita V.W., Marino P.D., Lucas P.A. (2016). The Distribution of Ochronosis in Osteoarthritic Articular Cartilage in a Knee. HSS J..

